# Disease Characteristics and Outcomes of 493 Young Myeloma Patients Treated With Modern Therapies: A Canadian Myeloma Research Group Database Study

**DOI:** 10.1002/cam4.70332

**Published:** 2024-11-05

**Authors:** Mégane Tanguay, Jean Roy, Jiandong Su, Engin Gul, Donna Reece, Christopher P. Venner, Darrell White, Michael P. Chu, Victor H. Jimenez‐Zepada, Kevin Song, Arleigh Mccurdy, Hira Mian, Michael Sebag, Debra Bergstrom, Julie Stakiw, Anthony Reiman, Rami Kotb, Muhammad Aslam, Rayan Kaedbey, Martha Louzada, Richard LeBlanc

**Affiliations:** ^1^ Institut universitaire d'hémato‐oncologie et de thérapie cellulaire Hôpital Maisonneuve‐Rosemont Montréal Québec Canada; ^2^ Université de Montréal Montréal Québec Canada; ^3^ Canadian Myeloma Research Group Vaughan Ontario Canada; ^4^ Princess Margaret Cancer Centre Toronto Ontario Canada; ^5^ BC Cancer – Vancouver Centre University of British Columbia Vancouver British Columbia Canada; ^6^ Queen Elizabeth II Health Sciences Centre Dalhousie University Halifax Nova Scotia Canada; ^7^ Cross Cancer Institute University of Alberta Edmonton Alberta Canada; ^8^ Arnie Charbonneau Cancer Institute University of Calgary Calgary Alberta Canada; ^9^ BC Cancer Agency Vancouver General Hospital Vancouver British Columbia Canada; ^10^ The Ottawa Hospital Ottawa Ontario Canada; ^11^ Juravinski Cancer Center Hamilton Ontario Canada; ^12^ Department of Medicine McGill University Montréal Québec Canada; ^13^ Memorial University of Newfoundland St John's Newfoundland and Labrador Canada; ^14^ Saskatoon Cancer Centre University of Saskatchewan Saskatoon Saskatchewan Canada; ^15^ Department of Oncology Saint John Regional Hospital Saint John New Brunswick Canada; ^16^ Cancer Care Manitoba Winnipeg Manitoba Canada; ^17^ Allan Blair Cancer Centre Regina Saskatchewan Canada; ^18^ Jewish General Hospital Montréal Québec Canada; ^19^ London Regional Cancer Center London Ontario Canada

**Keywords:** chemotherapy, clinical observations, epidemiology, multiple myeloma, registries, survival

## Abstract

**Background:**

Young patients ≤ 50 years old with multiple myeloma (MM) account for about 10% of cases and are underrepresented in the literature.

**Methods:**

We explored disease characteristics, treatments, and outcomes following modern therapies of young MM patients using the Canadian Myeloma Research Group (CMRG) database. We included 493 patients ≤ 50 years old diagnosed with MM or plasma cell leukemia without concurrent amyloidosis or POEMS syndrome from January 1, 2010, to July 1, 2022.

**Results:**

The median age was 46 years old (range: 25.6–50). Most patients fell into the R‐ISS II category (72.7%), and 24.1% had high‐risk cytogenetics. The majority of patients (89.9%) received a proteasome inhibitor‐based first‐line treatment, 92.1% received a stem cell transplant, and 65.6% had maintenance therapy post–autologous stem cell transplant (ASCT). Median follow‐up from initial treatment to patients' last follow‐up was 48.5 (range: 0–155) months. Median progression‐free survival (PFS) was 45.0 months (95% CI: 40.2–50.0). Maintenance therapy post‐ASCT improved median PFS to 52.3 months (95% CI: 43.1–68.2), compared to 23.6 months (95% CI: 20.0–34.8) without maintenance [*p* < 0.001].

**Conclusion:**

Although the overall survival has not yet been reached in this young population, our reported median PFS of only 45 months highlights the urgent need to develop innovative treatments to induce more profound and durable responses.

## Graphical abstract

We conducted a retrospective study using the Canadian Myeloma Research Group Database to explore disease characteristics, treatments, and outcomes of 493 patients with a median age of 46 years (range: 25.6–50). Although the overall survival has not yet been reached in this young population, our reported median PFS of only 45 months (95% CI: 40.2–50.0) highlights the urgent need to develop innovative treatments to induce more profound and durable responses.

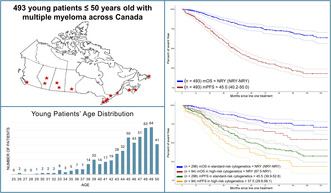

## 1 Introduction

Multiple myeloma (MM) predominantly impacts older adults, with a median age at diagnosis of 70 years [1, 2]. Despite significant recent therapeutic advances in treatments, MM remains largely incurable. The primary objectives of current therapies therefore focus on prolonging survival, alleviating symptoms, reversing or preventing organ damage, and improving quality of life. Although younger patients, defined herein as ≤ 50 years of age, represent approximately 10% of all cases, they experience the highest number of years lost due to their disease [3]. MM in young individuals also occurs during their peak productive years, leading to an amplified toll on personal, familial, professional, and financial burdens [4]. Young MM patients are currently underrepresented in clinical trials due to small numbers overshadowed in cohorts of older patients. Therefore, their disease characteristics and outcomes following modern treatments remain poorly described [5]. We aimed to bridge this gap by reporting disease characteristics, treatments, and outcomes of young MM patients treated in Canada with modern therapies and identifying prognostic factors related to disease progression and survival.

## 2 Methods

### 2.1 Patient Selection

We conducted a retrospective study using the Canadian Myeloma Research Group (CMRG) Database, a repository of real‐world patient data reflecting 17 of the major MM academic centers across Canada. This database contains comprehensive longitudinal disease‐specific outcome data on over 9000 patients added prospectively. It is rigorously maintained across institutions with monitoring checks to ensure data accuracy. The participation of all individuals in this study is contingent upon their informed consent by the Research Ethics Board (REB) requirements of each participating institution. All patients diagnosed with MM and/or plasma cell leukemia (PCL) without concurrent amyloidosis or POEMS syndrome from January 1, 2010, to July 1, 2022, were included. Patients who had not initiated first‐line treatment were excluded.

The following data at diagnosis were retrieved from the CMRG database: age, sex, year of diagnosis, previous history of monoclonal gammopathy of undetermined significance (MGUS) and smoldering myeloma, myeloma isotype, PCL, international staging system (ISS) and revised ISS (R‐ISS) stages, lytic bone lesions, creatinine level, hypercalcemia, lactate dehydrogenase (LDH), albumin, β2‐microglobulin, hemoglobin, neutrophil count, platelet count, and cytogenetics. Cytogenetic analyses were performed using fluorescence in situ hybridization (FISH) according to local protocols of individual centers. High‐risk cytogenetics were defined as del17p, t(4;14), and/or t(14;16). First, second, and third lines of treatment, type of transplant, if any, dates of progression and death, and causes of death were also collected.

### 2.2 Statistical Analysis

Patients' demographics, disease characteristics, treatments, and responses were collected for descriptive analyses. Patients were further divided into two groups: those ≤ 40 years old and those between 41 and 50 years old to compare baseline characteristics and cytogenetics. The age distribution of patients is shown in Figure S1.

The median and range were calculated for continuous variables. The percentages were determined for categorical variables based on patients with nonmissing values. *P*‐values for continuous variables were obtained using Mann–Whitney U tests or t‐tests depending on the variable distribution on the histogram plots, while *p*‐values for binary variables were determined through Chi‐square tests or Fisher's exact tests as appropriate.

Progression‐free survival (PFS) was defined as the start of the first‐line treatment to the earliest documented disease progression or death. Patients who did not experience the event of interest or were lost to follow‐up were censored at their last follow‐up. Overall survival (OS) was defined as the time from the start of the first‐line treatment to death. Patients who were still alive or lost to follow‐up were censored at the date of their last follow‐up. The median PFS and OS were estimated using the Kaplan–Meier method and the impact of covariates of interest was assessed using the log‐rank test. The timeframe considered spanned from the date of initial MM treatment to the date of disease progression, death, or the last follow‐up, whichever occurred first. We opted to use the date of initial treatment as our starting point instead of the diagnosis date due to database accuracy and better documentation of treatment start dates.

The impact of each patient's disease characteristics and treatment variables on survival was assessed using a Cox regression model. Variables included in univariable analysis were the year of diagnosis, age, biological sex, MM isotype, revised ISS stage, lytic bone lesions, serum creatinine, hypercalcemia, Bence Jones proteinuria, low hemoglobin, low platelets, the presence of PCL, cytogenetic risk, and use of stem cell transplant. Variable selection in multivariable analysis was based on clinical relevance and statistical fitting. Hazard ratios (HR) with 95% confidence intervals were estimated.

## 3 Results

### 3.1 Patients' Characteristics

Within the CMRG database between January 1, 2010 and July 1, 2022, 6594 patients were diagnosed with MM and/or PCL, after excluding those with concurrent amyloidosis or POEMS syndrome. Initially, 510 patients ≤ 50 years old were identified, but 17 were excluded due to not starting a first‐line treatment. Thus, 493 patients were included in the study, with 85 (1.3%) aged ≤ 40 years and 408 (6.2%) patients aged between 41 and 50. The patients' selection algorithm is shown in Figure S2.

Detailed demographics and disease characteristics at time of MM diagnosis are shown in Table 1. The median age of all patients ≤ 50 was 46 years (25.6–50), and male patients represented 58.2% of the cohort. The patients' characteristics did not show significant differences when comparing the age groups of ≤ 40 to those aged 41–50. Regarding MM isotype, immunoglobulin G was the most common in 50.1% of cases, while 22.5% had light‐chain MM. PCL was diagnosed in 2.4% of patients. Risk stratification revealed that most patients were R‐ISS II (72.7%), while 16.2% were R‐ISS I and 11.1% were R‐ISS III. As for myeloma‐defining events, 19% had hypercalcemia (> 2.65 mmol/L), 15.9% had elevated creatinine (> 177 μmol/L), 44.8% had anemia (< 100 g/L), and 46.9% had bone lytic lesions.

**TABLE 1:** Young patients' characteristics with percentages calculated among available data.

Patients' characteristics	18–40 years *n* (%)	41–50 years *n* (%)	*p‐value*	All patients *n* (%)
Number of patients	85 (17.2)	408 (82.8)		493 (100)
Year of diagnosis				
2010–2015	48 (56.5)	248 (60.8)	0.460	296 (60.0)
2016–2022	37 (43.5)	160 (39.2)		197 (40.0)
Median age, [range]	37.7 [25.6, 40.4]	46.9 [40.5, 50.0]	< 0.001	46.0 [25.6, 50.0]
Male sex	43 (50.6)	244 (59.8)	0.117	287 (58.2)
Previous history of MGUS	0 (0)	2 (0.5)	1.000	2 (0.4)
Previous history of smoldering MM	5 (5.9)	9 (2.2)	0.075	14 (2.8)
Type of myeloma				
IgG	44 (51.8)	203 (49.8)	0.736	247 (50.1)
IgA	17 (20.0)	98 (24.0)	0.425	115 (23.3)
IgM	0 (0)	2 (0.5)	1.000	2 (0.4)
IgD	3 (3.5)	3 (0.7)	0.067	6 (1.2)
Light‐chain only	18 (21.2)	93 (22.8)	0.745	111 (22.5)
Nonsecretory	3 (3.5)	9 (2.2)	0.443	12 (2.4)
Plasma cell leukemia	1 (1.2)	11 (2.7)	0.963	12 (2.4)
ISS stage	*n = 70*	*n = 324*		*n = 394*
I	29 (41.4)	108 (33.3)	0.197	137 (34.8)
II	23 (32.9)	113 (34.9)	0.747	136 (34.5)
III	18 (25.7)	103 (31.8)	0.318	121 (30.7)
R‐ISS stage	*n = 67*	*n = 303*		*n = 370*
I	16 (23.9)	44 (14.5)	0.006	60 (16.2)
II	41 (61.2)	228 (75.2)	0.019	269 (72.7)
III	10 (14.9)	31 (10.2)	0.268	41 (11.1)
Lytic bone lesions	*n = 61*	*n = 265*		*n = 326*
	31 (50.8)	122 (46.0)	0.500	153 (46.9)
Serum creatinine > 177 μmol/L	*n = 70*	*n = 371*		*n = 441*
	12 (17.1)	58 (15.6)	0.751	70 (15.9)
Hypercalcemia > 2.65 mmol/L	*n = 70*	*n = 335*		*n = 405*
	13 (18.6)	64 (19.1)	0.356	77 (19.0)
LDH above normal	*n = 62*	*n = 287*		*n = 349*
	8 (12.9)	54 (18.8)	0.269	62 (17.8)
Median albuminemia, g/L [range]	*n = 70*	*n = 351*		*n = 421*
	37.5 [16.0, 51.0]	36.0 [17.0, 52.0]	0.367	37.0 [16.0, 52.0]
Median β2‐microglobulin, nmol/L [range]	*n = 63*	*n = 322*		*n = 385*
	262 [104, 2730]	294 [76.3, 3710]	0.472	286 [76.3, 3710]
Hemoglobin < 100 g/L	*n = 73*	*n = 380*		*n = 453*
	34 (46.6)	169 (44.5)	0.741	203 (44.8)
Neutrophils < 1.0 × 10^9^/L	*n = 62*	*n = 283*		*n = 345*
	7 (11.3)	26 (9.2)	0.610	33 (9.6%)
Platelets < 50 × 10^9^/L	*n = 69*	*n = 339*		*n = 408*
	3 (4.4)	4 (1.2)	0.100	7 (1.7%)

Abbreviations: MGUS, monoclonal gammopathy of undetermined significance; MM, multiple myeloma; Ig, immunoglobulin; ISS, international staging system; R‐ISS, revised‐ISS; LDH, lactate dehydrogenase.

### 3.2 Cytogenetics

In patients ≤ 50 years old, 24.1% had high‐risk cytogenetics (Table 2). Specifically, 11.7% had del17p, 15.1% had t(4;14), and 5.2% had t(14;16). Fewer patients were tested for chromosome 1 abnormalities; of those, 24 of 85 (28.2%) had a 1q+ anomaly, while 11 of 71 (15.5%) had a 1p‐ anomaly. Among patients tested with at least three FISH probes (80 patients), 26.3% showed at least two cytogenetic anomalies. We observed no difference in cytogenetic abnormalities when comparing patients aged 18–40 versus 41–50 years old.

**TABLE 2:** Young patients' cytogenetics with percentages calculated among available data.

Patients' characteristics	18–40 years	41–50 years	*p‐value*	All patients
Number of patients	85 (17.2)	408 (82.8)		493 (100)
del17p				
Test performed	*n* = 60	*n* = 283		*n* = 343
Positivity; *n* (%)	10 (16.7)	30 (10.6)	0.356	40 (11.7)
t(4;14)				
Test performed	*n* = 56	*n* = 276		*n* = 332
Positivity; *n* (%)	6 (10.7)	44 (15.9)	0.589	50 (15.1)
t(14;16)				
Test performed	*n* = 42	*n* = 208		*n* = 250
Positivity; n (%)	1 (2.4)	12 (5.8)	0.771	13 (5.2)
1q+				
Test performed	*n* = 17	*n* = 68		*n* = 85
Positivity; *n* (%)	5 (29.4)	19 (27.9)	0.908	24 (28.2)
1p−				
Test performed	*n* = 14	*n* = 57		*n* = 71
Positivity; *n* (%)	2 (14.3)	9 (15.8)	0.956	11 (15.5)
Any high risk^a^				
Test performed	*n* = 67	*n* = 323		*n* = 390
Positivity; *n* (%)	16 (23.9)	78 (24.1)	0.963	94 (24.1)
≥ 3 FISH probes tested	*n* = 14	*n* = 66		*n* = 80
≥ 2 Anomalies; *n* (%)	4 (28.6)	17 (25.8)	1.000	21 (26.3)
< 2 Anomalies; *n* (%)	10 (71.4)	49 (74.2)		59 (73.8)

^a^ Either del17p or t(4;14) and/or t(14;16) present.

### 3.3 Treatments

Among the 493 young patients included in this study, all underwent at least one line of treatment in accordance with our inclusion criteria (Table 3). Within this cohort, MM was diagnosed in 296 patients (60%) between 2010 and 2015 and in 197 (40%) between 2016 and 2022. Most patients (89.9%) were treated with a proteasome inhibitor (PI)‐based chemotherapy as a first‐line regimen. Within this PI‐based group, the CyBorD regimen, comprising cyclophosphamide, bortezomib, and dexamethasone, was the predominant choice for 81.1% of patients. Only 6.7% of patients received an immunomodulatory (IMiD)‐based regimen, either alone or in combination with a PI. Subsequent lines of chemotherapy are described in Tables S1–S3.

**TABLE 3:** First‐line treatments in 493 young patients treated between January 2010 and July 2022.

	*n* = 493 (%)
PI‐based	443 (89.9)
CyBor+/−D/P^a^	400 (81.1)
VD/VP	40 (8.1)
VD‐PACE	1 (0.2)
VMP	2 (0.4)
IMiD‐based	18 (3.7)
R+/−D	17 (3.4)
T+/−D	1 (0.2)
PI + IMiD‐based	15 (3.0)
IxaR+/−D	2 (0.4)
KRD^b^	2 (0.4)
VRD	9 (1.8)
VTD	2 (0.4)
Other	17 (3.4)
MP	1 (0.2)
D‐PACE	1 (0.2)
VAD	1 (0.2)
Dexamethasone	14 (2.8)

Abbreviations: PI, proteasome inhibitor; CyBorD/P, cyclophosphamide + bortezomib + dexamethasone or prednisone; V, bortezomib; D, dexamethasone; P, prednisone; VD‐PACE, bortezomib + dexamethasone + cisplatin + doxorubicin + cyclophosphamide + etoposide; M, melphalan; IMiD, immunomodulatory drug; R, lenalidomide; T, thalidomide; Ixa, ixazomib; K, carfilzomib; D‐PACE, dexamethasone + cisplatin + doxorubicin + cyclophosphamide + etoposide; VAD, vincristine + doxorubicin + dexamethasone.

^a^ CyBorD + adriamycin (1 patient) + isatuximab (2 patients) + liposomal doxorubicin (1 patient).

^b^ KRD + daratumumab (1 patient).

In this study, most patients (92.1%) underwent stem cell transplantation during their treatment course (Table 4). The vast majority (90%) of the cohort underwent autologous stem cell transplantation (ASCT), not combined with allogeneic stem cell transplant (allo‐SCT). Within the ASCT subgroup, the majority (67.1%) received a single transplant. Meanwhile, 11.4% underwent tandem ASCT, and 11.4% received a second ASCT at the time of disease progression. Conversely, a minority of patients were treated with allo‐SCT. Among them, 1.6% underwent both autologous and allo‐SCT, and 0.6% received exclusively an allo‐SCT. Of the remaining 7.9% of patients who never received transplant, reasons included early death (2%), being lost to follow‐up (2.6%), being considered a poor transplant candidate or patient refusal (3%), and participation in a clinical trial excluding transplant (0.2%).

**TABLE 4:** Summary of stem cell transplant history and maintenance treatments among patients who received transplants.

	*n* = 493 (%)
Received stem cell transplant	454 (92.1)
Transplant type	
Autologous only	443 (90.0)
Upfront single ASCT	331 (67.1)
Tandem ASCT	56 (11.4)
Two ASCTs with second at disease progression	56 (11.4)
Autologous and allogeneic	8 (1.6)
Allogeneic only	3 (0.6)
Did not receive transplant	39 (7.9)
Reasons	
Early death before autologous transplant	10 (2.0)
Not a candidate/declined	15 (3.0)
Lost to follow‐up	13 (2.6)
Clinical trials excluding transplant	1 (0.2)
Maintenance post‐ASCT	*n* = 451 (%)
Received maintenance	296 (65.6)
Lenalidomide	250 (84.5)
Thalidomide	2 (0.7)
Ixazomib	3 (1.0)
Ixazomib + Lenalidomide +/− Dexamethasone	19 (6.4)
Bortezomib	7 (2.4)
Lenalidomide + Bortezomib +/− Dexamethasone	7 (2.4)
Pomalidomide + Bortezomib + Dexamethasone	1 (0.3)
Pomalidomide +/− Dexamethasone	2 (0.7)
Carfilzomib + Lenalidomide + Dexamethasone	2 (0.7)
Isatuximab	1 (0.3)
Isatuximab + Lenalidomide	2 (0.7)
Did not receive maintenance post‐ASCT	155 (34.4)
Reasons	
Received consolidation	1 (0.6)
Early relapse or death prior to start of maintenance	101 (65.2)
Declined	3 (1.9)
Unknown/lost to follow‐up	50 (32.2)

Abbreviation: ASCT, autologous stem cell transplant.

Among patients who underwent ASCT, 65.6% received maintenance therapy post‐transplant, with lenalidomide accounting for 84.5% of these treatments (Table 4). In contrast, 34.4% of patients did not undergo maintenance therapy. Several factors contributed to the omission of maintenance therapy, including consolidation treatment (0.6%), early death or relapse (65.2%), patient refusal (1.9%), and other reasons such as unspecified cases or loss of follow‐up (32.2%). Within the unspecified category, it is noteworthy that some patients underwent transplantation before 2013, a period when maintenance therapy was not standard practice in Canada. In the Canadian healthcare system, each province has the authority to decide which treatments are approved. The adoption of maintenance therapy became more widespread post‐2013, following studies that endorsed its use, particularly highlighting the efficacy of lenalidomide [6, 7]. In our cohort, only 50% of patients diagnosed between 2010 and 2013 received maintenance therapy, compared to 72% of those diagnosed between 2014 and 2022 (*p* < 0.001).

### 3.4 Responses and Survival

Following induction therapy, the overall response rate (ORR), which includes partial response (PR) or better, was 95.6%, with 59.9% of patients attaining at least a very good partial response (VGPR) (Table S4a). The ORR after stem cell transplantation was 97.8%, with 82.6% achieving VGPR or better (Table S4b).

**FIGURE 1:**
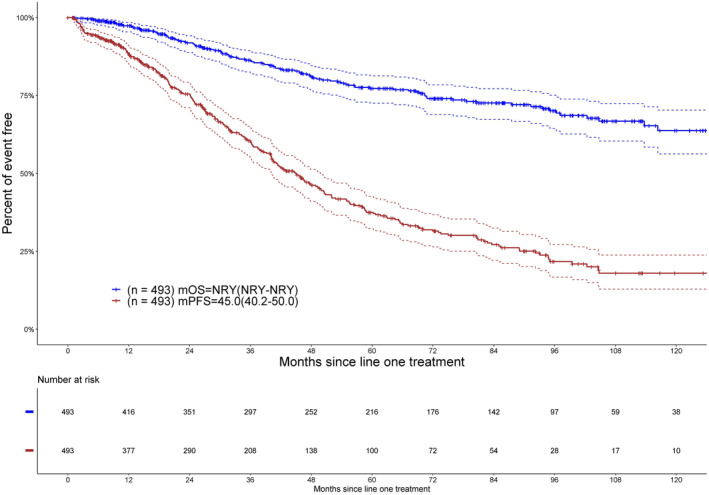
Shown are overall survival (OS) and progression‐free survival (PFS) in months of young patients diagnosed between 2010 and 2022. m, median; NRY, not reached yet.

Survival data were evaluated for the entire cohort of 493 patients. Median follow‐up from initial treatment to patients' last follow‐up was 48.5 (range: 0–155) months. The median PFS following initial treatment was 45.0 months (95% CI: 40.2–50.0) (Figure 1). The median OS was not reached during the study period (Figure 1). At 5 years, the PFS was 37% (95% CI: 33–43) and the OS was 77% (95% CI: 73–82). At 10 years, the PFS was 18% (95% CI: 13–24), and the OS was 64% (95% CI: 57–71). During the follow‐up period, 104 patients (21.1%) died. The primary causes of death were disease progression in 83.6%, treatment toxicity in 2.9%, and unspecified reasons in 13.5%.

**FIGURE 2:**
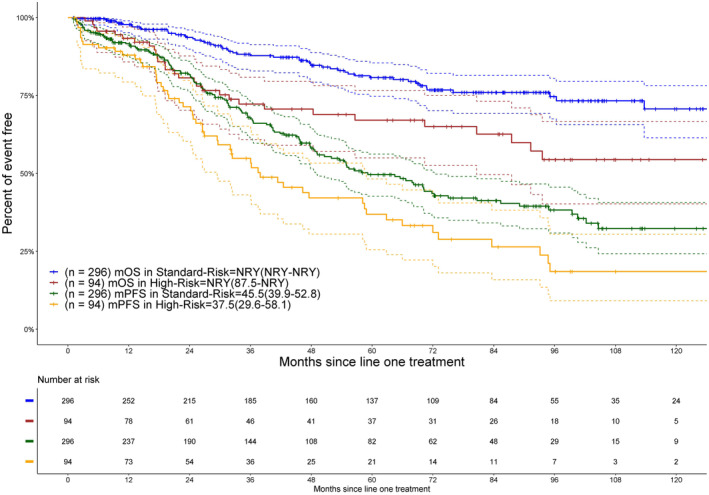
Shown are overall survival (OS) and progression‐free survival (PFS) in months of young patients based on cytogenetic risk. m, median; NRY, not reached yet.

Survival was evaluated per cytogenetic risk in 390 patients with available data (Figure 2). The median PFS was shorter for patients with high‐risk cytogenetics at 37.5 months (95% CI: 29.6–58.1), compared to 45.5 months (95% CI: 39.9–52.8) for those with standard‐risk cytogenetics, approaching statistical significance with a *p*‐value of 0.0571. The median OS stratified by cytogenetic risk was not reached in both subgroups, yet it was significantly poorer in high‐risk patients (*p* = 0.0015).

Survival based on R‐ISS was also performed in 370 patients with available data. The median PFS for R‐ISS I, R‐ISS II, and R‐ISS III patients was 55.4 (95% CI: 44.5–not reached), 42.2 (95% CI: 37.9–50), and 36.2 months (95% CI: 26.4–94.7), respectively. The variation in PFS across the different R‐ISS groups was statistically significant (*p* = 0.0187; Figure S3a). The median OS was not reached for any R‐ISS categories (Figure S3b). However, there was a statistically significant difference in survival probabilities among these groups (*p* = 0.0066).

**FIGURE 3:**
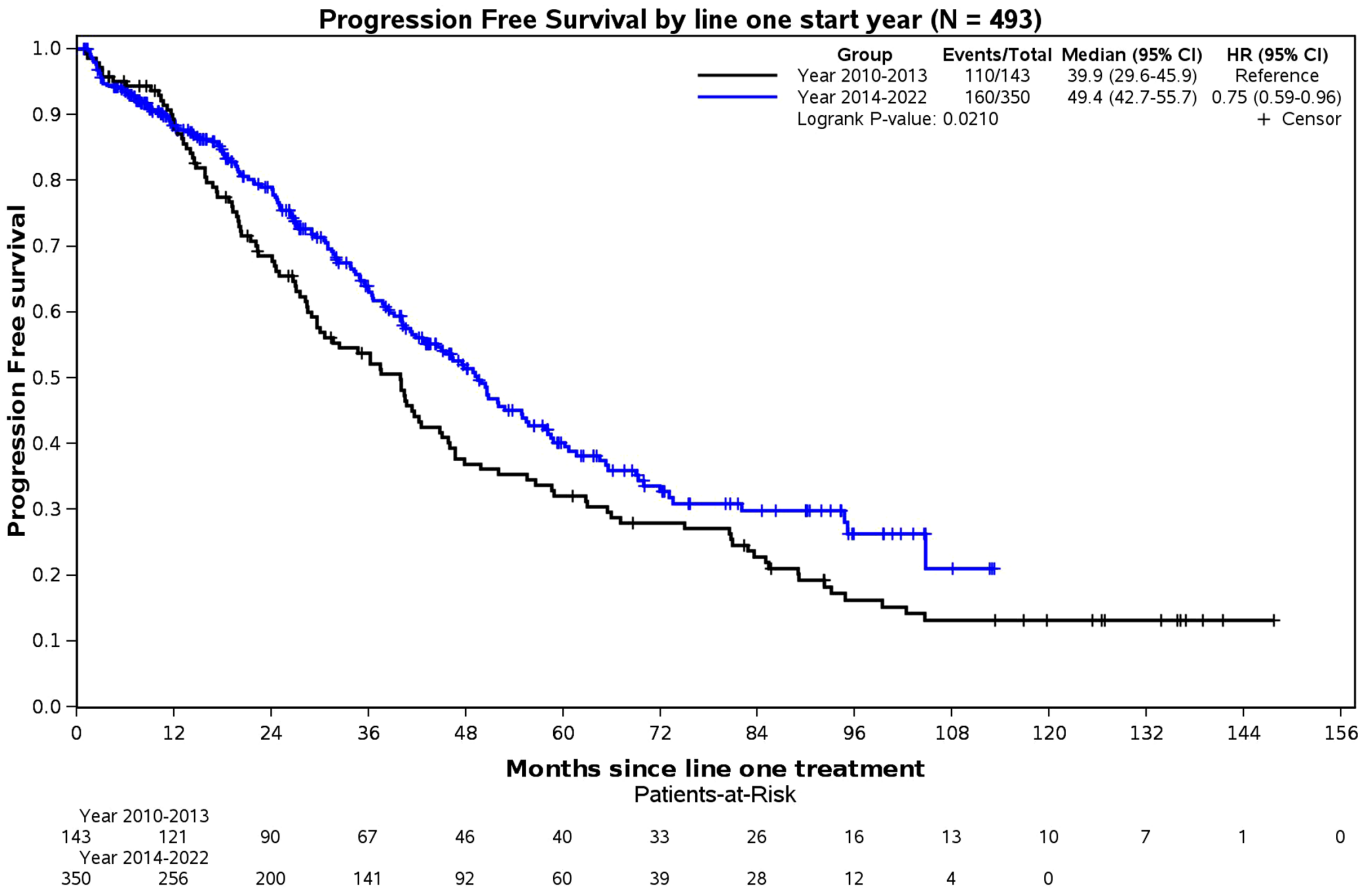
Progression‐free survival (PFS) comparing patients diagnosed between 2010 and 2013 to patients diagnosed between 2014 and 2022.

When comparing 2010 to 2013 and 2014 to 2022, we observed a statistically significant increase in median PFS. Patients treated between 2014 and 2022 had a median PFS of 49.4 months (95% CI: 42.7–55.7), in contrast to those treated from 2010 to 2013, who had a median PFS of 39.9 months (95% CI: 29.6–45.9) [*p* = 0.0210] (Figure 3). Analysis of cohorts based on sex and age groups ≤ 40 versus 41–50 years old revealed no significant differences in outcomes (Figures S4 and S5).

**FIGURE 4:**
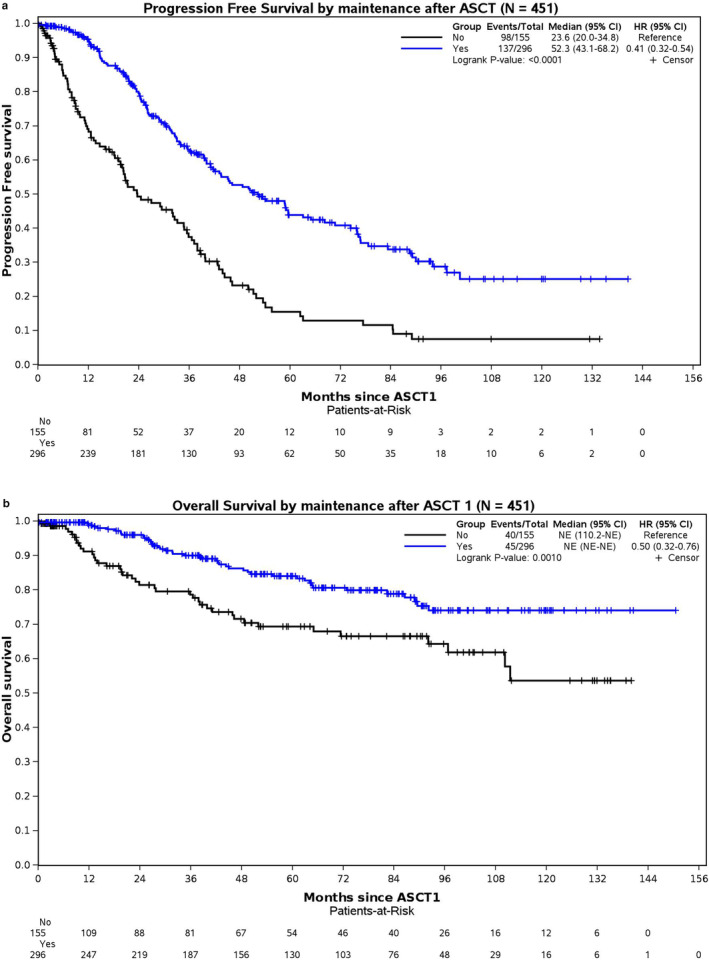
(a) Progression‐free survival (PFS) based on maintenance or no maintenance after autologous stem cell transplant (ASCT). (b) Overall survival (OS) based on maintenance or no maintenance after autologous stem cell transplant (ASCT).

When comparing patients who received maintenance therapy post‐ASCT with those who did not, both median PFS and OS showed a significant benefit for patients who received maintenance treatment. Specifically, patients undergoing maintenance therapy experienced a median PFS of 52.3 months (95% CI: 43.1–68.2), significantly longer than the 23.6 months (95% CI: 20.0–34.8) observed in patients who did not receive maintenance, with a *p* < 0.001 (Figure 4a). While the median OS for patients who received maintenance has not been reached, the comparison still revealed statistically significant improvements (*p* = 0.001; Figure 4b).

### 3.5 Prognostic Factors for Response and Outcomes

The impact of each patient's disease characteristics and treatment variables on progression and survival was assessed using univariable Cox regression. Only clinically relevant data from the univariable analysis are presented in Table S5a,b, while results from the multivariable analysis are detailed in Table S6a,b. In the univariable analysis, patients with high‐risk cytogenetics showed a trend toward an increased likelihood of disease progression (HR 1.34, 95% CI: 1.00–1.81; *p* = 0.054) and a significantly increased risk of death (HR 2.08, 95% CI: 1.33–3.26; *p* = 0.001). This association was not significant in the multivariable analysis for disease progression (HR 1.21, 95% CI: 0.89–1.64; *p* = 0.224) but significant for the outcome of death (HR 1.88, 95% CI: 1.19–2.97; *p* = 0.007).

Elevated LDH levels were also correlated with a higher risk of disease progression (HR 1.65, 95% CI: 1.17–2.34; *p* = 0.004) and a higher risk of death (HR 3.03, 95% CI: 1.88–4.89; *p* < 0.001) in the univariable model. In the multivariable model, there was a trend toward significance for disease progression (HR 1.41, 95% CI: 0.97–2.04; *p* = 0.068), and there was an increase in the risk of death (HR 2.20, 95% CI: 1.29–3.74; *p* = 0.004) with higher levels of LDH.

Patients who underwent transplantation had a significantly reduced risk of disease progression (HR 0.37, 95% CI: 0.25–0.56; *p* < 0.001) and death (HR 0.22, 95% CI: 0.13–0.36; *p* < 0.001) in the univariable analysis. This remained significant in the multivariable analysis for both disease progression (HR 0.41, 95% CI: 0.26–0.65; *p* < 0.001) and death (HR 0.29, 95% CI: 0.16–0.54; *p* < 0.001).

Surprisingly, compared to stage I, ISS stages II and III did not reach statistical significance in univariable and multivariable analyses for disease progression and death. Patients with PCL had an increased risk of death in the univariable analysis (HR 2.31, 95% CI: 1.01–5.26, *p* = 0.047), but not in the multivariable analysis. Both univariable and multivariable analyses for outcome of disease progression showed no statistically significant increased risk in patients with PCL.

## 4 Discussion

MM is infrequently diagnosed in patients ≤ 50 years old. The available literature lacks detailed descriptions of their disease characteristics, treatments, and outcomes. Our study reports one of the largest cohorts of young MM patients treated with modern therapies in a public healthcare system with universal access, comprising 493 patients aged ≤ 50.

In our study, the incidence of light‐chain disease was marginally elevated at 22.5%, compared to a prevalence of approximately 15% in the general myeloma population [8]. This finding is consistent with other studies that have reported a higher rate of light‐chain disease in younger MM patients [9, 10, 11, 12, 13, 14, 15, 16]. Additionally, we observed a higher incidence of ISS I specifically in the 18–40 age group, which aligns with previous studies reporting a predominance of low ISS among young MM patients [10, 12, 14, 15, 17, 18, 19, 20, 21]. High‐risk cytogenetics were present in 24.1% of our cohort, comparable to the general MM population [22]. While there is significant heterogeneity in the incidence of high‐risk cytogenetics among young MM cohorts [5], some studies have reported a greater prevalence of high‐risk cytogenetics in younger patients [18]. Overall, when analyzing the different disease characteristics of our young cohort, we find no specific factor prominently distinct compared with older cohorts from the literature.

Within our young MM cohort, up to 40% received treatment between 2016 and 2022, indicating that a significant portion of our patients were treated with more modern regimens. The treatments received reflect the prevalent practices of the Canadian public healthcare system during the study period, where only approved treatments are publicly covered. As a result, most patients received CyBorD as induction therapy followed by maintenance, since it was the funded standard at the time. A minority received a PI and IMiD combination as first‐line treatment due to the lack of coverage by Canadian provinces at time of inclusion. However, in the last 2 years, the combination of bortezomib, lenalidomide, and dexamethasone (VRD) has been established as the preferred regimen for transplant‐eligible patients and is expected to rise among newly included patients in the CMRG database. Of note, no patients received quadruplet therapies as first‐ or second‐line treatment, as these treatments are not publicly covered. Unsurprisingly, more recent regimens for relapsed myeloma, which could favorably impact OS, such as chimeric antigen T‐cell therapy, T‐cell–engaging bispecific antibodies, and cereblon E3 ligase modulators, were rarely used in our cohort as they too were not approved in Canada during the study period. Transplantation was performed in up to 92.1% of patients during their treatment course, in keeping with our national consensus guidelines. The overall utilization of ASCT was similar to that of Caulier et al., who noted that 93% of their patients ≤ 40 years underwent transplant [14]. Although most of our patients received maintenance post‐ASCT, 34.4% did not, partly explained by the fact that maintenance therapy was evolving as a standard practice in Canada after 2013.

We observed an improvement in PFS for patients diagnosed between 2014 and 2022, with a median PFS of 49.4 months, compared to those diagnosed earlier between 2010 and 2013 who had a median PFS of 39.9 months. This trend of improved MM outcomes over time aligns with findings from other studies [16, 23]. The gain in PFS is likely attributed to implementing maintenance therapy following ASCT. Specifically, maintenance therapy markedly increased the median PFS from 23.6 months without maintenance to 52.3 months with maintenance within our young patient cohort. A significant OS benefit was also noted; although the median OS has not been reached, the survival analysis showed statistical significance. In our cohort, significantly more patients received post‐transplant maintenance when diagnosed between 2014 and 2022 versus 2010 and 2013 (72% vs. 50%, *p* < 0.001), contributing to the improved PFS in more recent years. These results align with the recent study by Côté et al., which also utilized the CMRG database [24].

We report a median PFS following initial treatment of 45.0 months, with a median OS not yet reached. At 5 years, the OS was 77% and 64% at 10 years. Our findings compare favorably with other retrospective studies in young patients. Caulier et al., who analyzed patients ≤ 40 years old between 2000 and 2015, documented a median PFS of 41 months and a 5‐year OS estimate of 84%, which fell to 59% by the 10‐year mark [14]. Furthermore, research targeting patients aged 40 or younger found 5‐year OS rates that align closely with our slightly older cohort [10, 18, 25].

The 45‐month median PFS observed in our young cohort underscores the need for more effective treatments. Young patients, capable of withstanding more aggressive therapies, stand to benefit from innovative approaches. Currently, in Canada, all transplant‐eligible patients (typically 70 years and younger) receive similar first‐line treatments regardless of their specific age. The lack of tailored treatments for patients aged 50 years or younger certainly negatively affects outcomes. The inclusion of quadruplet therapy with daratumumab, bortezomib, lenalidomide, and dexamethasone (D‐VRd) before transplant offers a promising avenue to enhance PFS. Evidence from the PERSEUS study suggests that younger patients particularly benefit from D‐VRd, likely due to their better tolerance to toxicities [26]. The notably higher PFS rate of 84.3% at 48 months, as opposed to 67.7% with VRD alone, reinforces the argument for its funding by our public healthcare system. Tandem autologous/allo‐SCT should also be considered in selected young patients with MM, as encouraging results have been published [27, 28].

Unsurprisingly, we found that high‐risk cytogenetics and elevated LDH negatively affected disease progression and survival, while ASCT reduced these risks. Conversely, the ISS stages did not statistically influence disease progression and mortality in univariable and multivariable analyses. We hypothesize that the ISS might not be a useful prognostic tool for younger MM patients. Cytogenetics, included in the R‐ISS, appears to play a more crucial role in outcomes in our cohort. The advancements in treatment modalities might render the ISS classification less relevant or outdated for younger MM patients. Also, a PCL diagnosis did not significantly affect disease progression and survival in multivariable analysis, most likely due to the small number of patients in this study.

Our study faces limitations due to its reliance on a patient registry. Firstly, this is a retrospective analysis subject to missing data and potential entry errors. Also, the lack of data on ethnicity, standardized maintenance duration, and clinic visits for disease assessment among patients may all have impacted the accuracy of survival outcomes such as PFS. Additionally, the different FISH‐positive cutoffs complicate the comparison of cytogenetic data across various centers. Despite these challenges, it is essential to recognize that the CMRG database is rigorously maintained prospectively. Proactive efforts were made to ensure data accuracy, including multiple queries to participating centers. Finally, our trial focused only on younger individuals and was not designed to compare the results with those over 50.

In summary, we report one of the largest real‐world cohorts of young MM patients ≤ 50 years old treated with modern therapies in a public healthcare system. The disease characteristics of our young cohort mirror those found in older patient cohorts from the literature. Our data also suggest that MM in younger patients may not represent a distinct entity but rather the same disease manifesting in individuals at an earlier age. Although the OS has not yet been reached in this young population, our reported median PFS of only 45 months highlights the urgent need to develop more effective treatments to induce more profound and more durable responses after induction and transplant. Monitoring the outcomes of young patients within a public healthcare system, especially as coverage policies evolve, is crucial. Our study is essential for enabling the comparison of outcomes in future young patients and serves as a benchmark for evaluating the effectiveness of newer treatments in Canada and worldwide. Furthermore, the suboptimal PFS observed in our nationwide study underscores the need to advocate for policy changes in drug coverage, potentially persuading public authorities to implement more effective and tailored treatments for young MM patients.

## Author Contributions

**Mégane Tanguay:** conceptualization (lead), formal analysis (lead), methodology (lead), writing – original draft (lead). **Jean Roy:** conceptualization (lead), methodology (lead), writing – review and editing (equal). **Jiandong Su:** data curation (lead), formal analysis (lead), writing – review and editing (equal). **Engin Gul:** project administration (lead), writing – review and editing (equal). **Donna Reece:** data curation (equal), writing – review and editing (equal). **Christopher P. Venner:** data curation (equal), writing – review and editing (equal). **Darrell White:** data curation (equal), writing – review and editing (equal). **Michael P. Chu:** data curation (equal), writing – review and editing (equal). **Victor H. Jimenez‐Zepada:** data curation (equal), writing – review and editing (equal). **Kevin Song:** data curation (equal), writing – review and editing (equal). **Arleigh Mccurdy:** data curation (equal), writing – review and editing (equal). **Hira Mian:** data curation (equal), writing – review and editing (equal). **Michael Sebag:** data curation (equal), writing – review and editing (equal). **Debra Bergstrom:** data curation (equal), writing – review and editing (equal). **Julie Stakiw:** data curation (equal), writing – review and editing (equal). **Anthony Reiman:** data curation (equal), writing – review and editing (equal). **Rami Kotb:** data curation (equal), writing – review and editing (equal). **Muhammad Aslam:** data curation (equal), writing – review and editing (equal). **Rayan Kaedbey:** data curation (equal), writing – review and editing (equal). **Martha Louzada:** data curation (equal), writing – review and editing (equal). **Richard LeBlanc:** conceptualization (lead), data curation (equal), methodology (lead), writing – review and editing (equal).

## Ethics Statement

This project was approved by the Maisonneuve‐Rosemont Hospital Ethics Committee.

## Conflicts of Interest

M.T. declares no competing interests. J.R. has received honoraria from Janssen and was part of advisory committees for Sanofi, Amgen, and Janssen. He received grant support and had royalties from ExCellThera. D.R. has received honoraria from BMS, Janssen, Takeda, Sanofi, Pfizer, and GSK. She served as a consultant for Janssen, Amgen, Takeda, and BMS. She received research funding from Janssen, Takeda, BMS, and Millennium. C.P.V. has received honoraria from Janssen, BMS, Pfizer, Abbvie, Sanofi, Forus, and GSK. D.W. has received honoraria from Amgen, Antengene, BMS, Forus, GSK, Janssen, Karyopharm, Pfizer, Sanofi, and Takeda. M.P.C. has received honoraria from AstraZeneca, BMS/Celgene, Gilead, Janssen, AbbVie, Pfizer, Sanofi, and Amgen. He received research funding from BMS/Celgene and Miltenyi. V.H.J‐Z. has received honoraria from Celgene, Janssen, Takeda, Merck, and BMS. K.S. has received honoraria from BMS, Janssen, Amgen, and Sanofi. A.M. has received honoraria from Celgene, Janssen, Amgen, Takeda, Sanofi, and GSK. H.M. has received honoraria from Celgene, Janssen, Amgen, Takeda, and Sanofi. She received the GSK Awards: HHS Research Early Career Award from the Hamilton Health Sciences Foundation. M.S. had membership on an entity's Board of Directors or advisory committees for Janssen, Amgen, Takeda, and Celgene. D.B. has received honoraria from Janssen and BMS. She received research funding from BMS. J.S. has received honoraria from Janssen, FORUS Therapeutics, BMS, Pfizer, Sanofi, and Amgen. A.R. served as a consultant and received honoraria and research funding from Janssen, Sanofi, BMS, Takeda, Pfizer, Regeneron, and AstraZeneca. R. Ko. has received honoraria from Akcea, Amgen, BMS, Janssen, Merck, Sanofi, Celgene, Pfizer, and Takeda. He has received research funding from Merck and Sanofi and holds equity in the private company Karyopharm. M.A. has received honoraria from AbbVie, Gilead, Janssen, and Celgene. R.Ka. has received honoraria from Janssen, BMS, FORUS, Sanofi, and Pfizer. M.L. has received honoraria from Janssen, Celgene, Amgen, and Pfizer. R.L. received research funding from Amgen and Sanofi, served as a consultant for BMS, Janssen, Amgen, Sanofi, and FORUS Therapeutics, and received honoraria from Pfizer and Janssen.

## Supporting information


Data S1.

## Data Availability

The datasets for the current study are not publicly available due to privacy regulations and the Canadian Myeloma Research Group database governance. They can be made available upon a reasonable request to the Canadian Myeloma Research Group.
