# Supplementary material for: Disease Characteristics and Outcomes of 493 Young Myeloma Patients Treated With Modern Therapies: A Canadian Myeloma Research Group Database Study
Source: Cancer Med. 2024 Nov 5;13(21):e70332. doi: 10.1002/cam4.70332 (PMC11536480; doi:10.1002/cam4.70332)
Supplement: Supplementary file 1 — Data S1. [file CAM4-13-e70332-s001.docx]

**SUPPLEMENTAL TABLES**

|  | *n = 141* (%) |
| --- | --- |
| PI-based  CyBorD  VD/VP | **24 (17.0)**  19 (13.5)  5 (3.5) |
| IMiD-based  R+/-D  T+/-D  Dara-RD | **97 (68.8)**  94 (66.7)  2 (1.4)  1 (0.7) |
| PI + IMiD-based  IxaR+/-D  KRD  VRD | **17 (12.1)**  7 (5.0)  1 (0.7)  9 (6.4) |
| Other  MP  Dexamethasone | **3 (2.1)**  1 (0.7)  2 (1.4) |

**Table S1. Second line treatments in 141 young myeloma patients treated between January 2010 and July 2022.** PI: proteasome inhibitor; CyBorD: cyclophosphamide + bortezomib + dexamethasone; V: bortezomib; P: prednisone; IMiD: immunomodulatory drug; R: lenalidomide; D: dexamethasone; T: thalidomide; Dara: daratumumab; Ixa: ixazomib; K: carfilzomib; M: melphalan.

| Third line treatments, n (%) | *n = 138 (%)* |
| --- | --- |
| PI-based  CyBorD  DaraKD  DaraVD  Ixa +/- D  IxaCD  IxaKD  KC +/- D  V +/- D  SelVD  VD-PACE  VD+ABT-888  VC+ISO906  Perifosine +V | **48 (34.8)**  15 (10.9)  2 (1.4)  5 (3.6)  5 (3.6)  2 (1.4)  1 (0.7)  4 (2.9)  5 (3.6)  2 (1.4)  3 (2.2)  2 (1.4)  1 (0.7)  1 (0.7) |
| IMiD-based  R +/- D  DaraRD  Pom +/- D  DaraPomD +/- C  Isatuximab + PomD  Elotuzumab + Pom +/- D  Elotuzumab + RD  BiRD | **64 (46.4)**  43 (31.2)  6 (4.3)  1 (0.7)  6 (4.3)  1 (0,7)  4 (2.9)  1 (0.7)  2 (1.4) |
| PI + IMiD-based  KRD  RVD  VTD-PACE  IxaR +/- D  IxaPomD  DaraPomVD | **22 (15.9)**  3 (2.2)  8 (5.8)  1 (0.7)  8 (5.8)  1 (0.7)  1 (0.7) |
| Other  DaraCD  CD  Dexamethasone | **4 (2.9)**  1 (0.7)  2 (1.4)  1 (0.7) |

**Table S2. Third line treatments in 138 young myeloma patients treated between January 2010 and July 2022.** PI: proteasome inhibitor; CyBorD: cyclophosphamide + bortezomib + dexamethasone; Dara: daratumumab; K: carfilzomib; D: dexamethasone; V: bortezomib; Ixa: ixazomib; C: cyclophosphamide; Sel: Selinexor; VD-PACE : bortezomib + dexamethasone + cisplatin + doxorubicin + etoposide; VP: bortezomib + prednisone; IMiD: immunomodulatory drug; R: lenalidomide; Pom: pomalidomide; BiRD: clarithromycin + lenalidomide + dexamethasone; VTD-PACE: bortezomib + thalidomide + dexamethasone + cisplatin + doxorubicin + cyclophosphamide + etoposide.

| Fourth line treatments, n (%) | *Valid data n = 116*  Value |
| --- | --- |
| PI-based  CYBORD  DaraKD  DaraVD  Ixa +/- D  IxaCD  K+/-C +/-D  VD  Isatuximab + KD | **28 (24.1)**  6 (5.2)  1 (0.9)  8 (6.9)  2 (1.7)  1 (0.9)  8 (6.9)  1 (0.9)  1 (0.9) |
| IMiD-based  R +/- D  RCD  DaraRD  Pom +/- C +/- D  DaraPomD +/- C  IsaPomDex  SelPomD  BCMA-AB drug conjugate  + PomDex | **50 (43.1)**  19 (16.4)  4 (3.4)  5 (4.3)  17 (14.7)  1 (0.9)  1 (0.9)  1 (0.9)  2 (1.7) |
| PI + IMiD-based  KRD  RVD  IxaRD  IxaPomD  IxaDaraRD  PomKC +/- D  PomVD  Vor + RVD + Biaxin | **26 (22.4)**  3 (2.6)  7 (6.0)  2 (1.7)  4 (3.4)  1 (0.9)  2 (1.7)  6 (5.2)  1 (0.9) |
| Other  Bispecific AB  CAR-T Cell  CELMoD  DaraD + CELMoD  D-PACE  DaraCD  Isatuximab | **12 (10.3)**  2 (1.7)  1 (0.9)  1 (0.9)  1 (0.9)  3 (2.6)  3 (2.6)  1 (0.9) |

**Table S3. Fourth line treatments in 116 young myeloma patients treated between January 2010 and July 2022.** PI: proteasome inhibitor; CyBorD: cyclophosphamide + bortezomib + dexamethasone; Dara: daratumumab; K: carfilzomib; D: dexamethasone; V: bortezomib; Ixa: ixazomib; C: cyclophosphamide; Pom: pomalidomide; Isa: isatuximab; Sel: Selinexor; BCMA-AB: B cell maturation antigen antibody; R: revlimid; Vor: vorinostat; CAR: chimeric antigen receptor; CELMoD: Cereblon E3 Ligase Modulatory Drugs; PACE: cisplatin + doxorubicin + cyclophosphamide + etoposide.

|  | *n = 411 (%)* |
| --- | --- |
| Overall response rate  PR and better | 393 (95.6) |
| Very good partial response or better  Including CR, NCR, MR, and VGPR | 246 (59.9) |
| Partial response | 147 (35.8) |
| Stable disease | 13 (3.2) |
| Progressive disease | 5 (1.2) |

**Table S4a. Best response after induction therapy before stem cell transplantation.** PR: partial response; CR: complete response; NCR: near complete response; MR: minimal response; VGPR: very good partial response.

|  | *n = 367 (%)* |
| --- | --- |
| Overall response rate  PR and better | 359 (97.8) |
| Very good partial response or better  Including CR, MR, NCR, VGPR | 303 (82.6) |
| Partial response | 56 (15.3) |
| Stable disease | 5 (1.4) |
| Progressive disease | 3 (0.8) |

**Table S4b. Best response after stem cell transplantation.** PR: partial response; CR: complete response; NCR: near complete response; MR: minimal response; VGPR: very good partial response.

|  | **Univariable Cox regression** | | | |
| --- | --- | --- | --- | --- |
| **Predictors** | **Hazard Ratio** | **Low 95% CI** | **Upper 95% CI** | **p-value** |
| **ISS stage** |  | | | |
| I | Reference | | | |
| II | **1.37** | 1.00 | 1.89 | 0.051 |
| III | **1.38** | 0.99 | 1.92 | 0.058 |
| Unknown | **1.18** | 0.82 | 1.69 | 0.378 |
| **Cytogenetic risk** |  | | | |
| Standard | Reference | | | |
| High | **1.34** | 1.00 | 1.81 | 0.054 |
| Not done | **0.94** | 0.69 | 1.28 | 0.701 |
| **LDH above normal value (280 U/L)** |  | | | |
| No | Reference | | | |
| Yes | **1.65** | 1.17 | 2.34 | 0.004 |
| Unknown | **1.30** | 0.99 | 1.71 | 0.058 |
| **Plasma cell leukemia** |  | | | |
| No | Reference | | | |
| Yes | **1.47** | 0.73 | 2.97 | 0.286 |
| **Had autologous or allogeneic stem cell transplant** |  | | | |
| No | Reference | | | |
| Yes | **0.37** | 0.25 | 0.56 | <0.001 |

**Table S5a. Cox proportional regression for the outcome of disease progression after first line of treatment (with clinician specified covariates).**

|  | **Univariable Cox regression** | | | |
| --- | --- | --- | --- | --- |
| **Predictors** | **Hazard Ratio** | **Low 95% CI** | **Upper 95% CI** | **p-value** |
| **ISS stage** |  | | | |
| I | Reference | | | |
| II | **1.23** | 0.73 | 2.08 | 0.437 |
| III | **1.41** | 0.82 | 2.42 | 0.208 |
| Unknown | **1.47** | 0.83 | 2.62 | 0.189 |
| **Cytogenetic risk** |  | | | |
| Standard | Reference | | | |
| High | **2.08** | 1.33 | 3.26 | 0.001 |
| Not done | **1.23** | 0.75 | 2.03 | 0.409 |
| **LDH above normal value (280 U/L)** |  | | | |
| No | Reference | | | |
| Yes | **3.03** | 1.88 | 4.89 | <0.001 |
| Unknown | **1.16** | 0.73 | 1.84 | 0.543 |
| **Plasma cell leukemia** |  | | | |
| No | Reference | | | |
| Yes | **2.31** | 1.01 | 5.26 | 0.047 |
| **Had autologous or allogeneic stem cell transplant** |  | | | |
| No | Reference | | | |
| Yes | **0.22** | 0.13 | 0.36 | <0.001 |

**Table S5b. Cox proportional regression for the outcome of death after first line of treatment (with clinician specified covariates).**

**
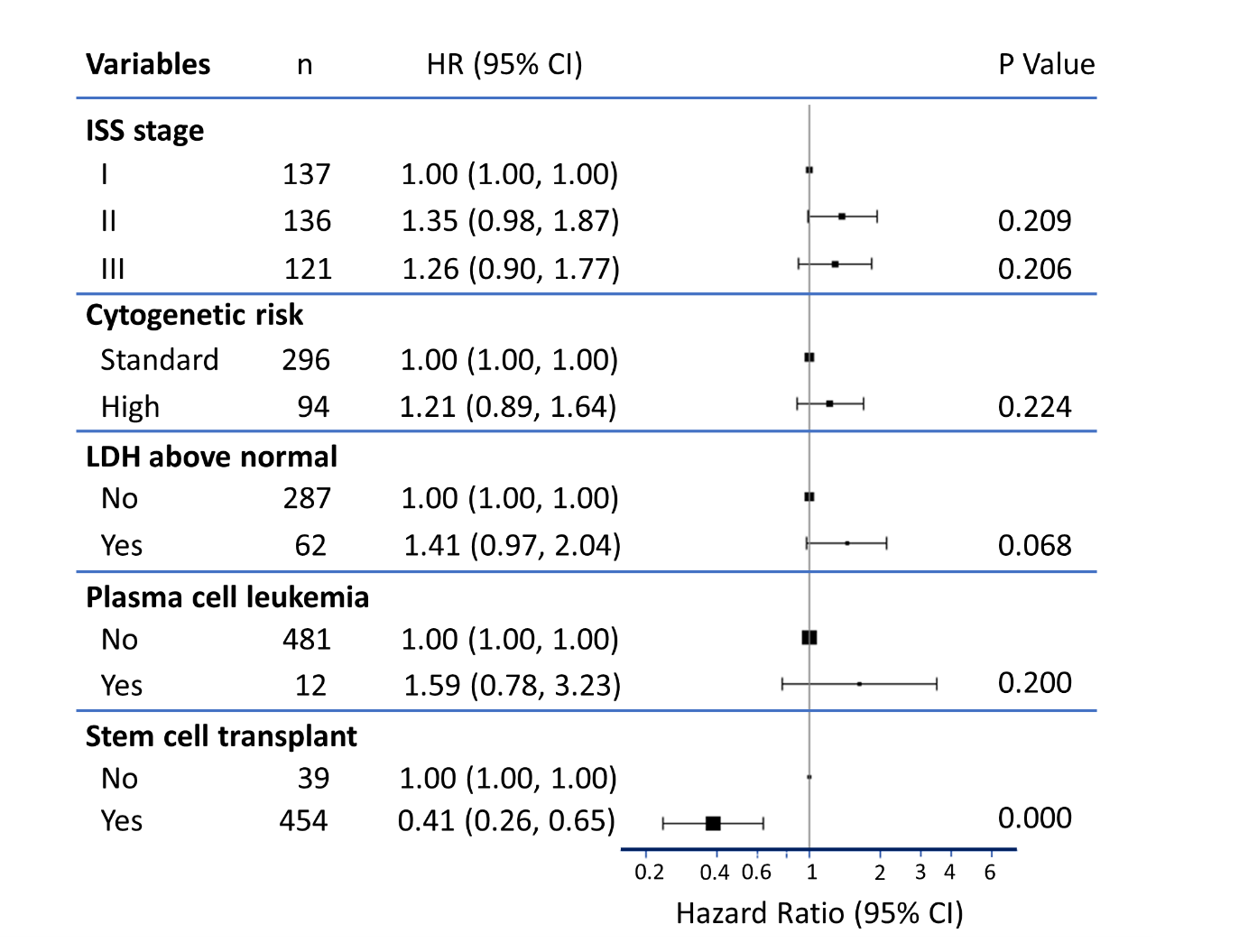
**

**Table S6a. Forest plot showing multivariable regression for the outcome of disease progression after first line of treatment (with clinician specified covariates).**

**
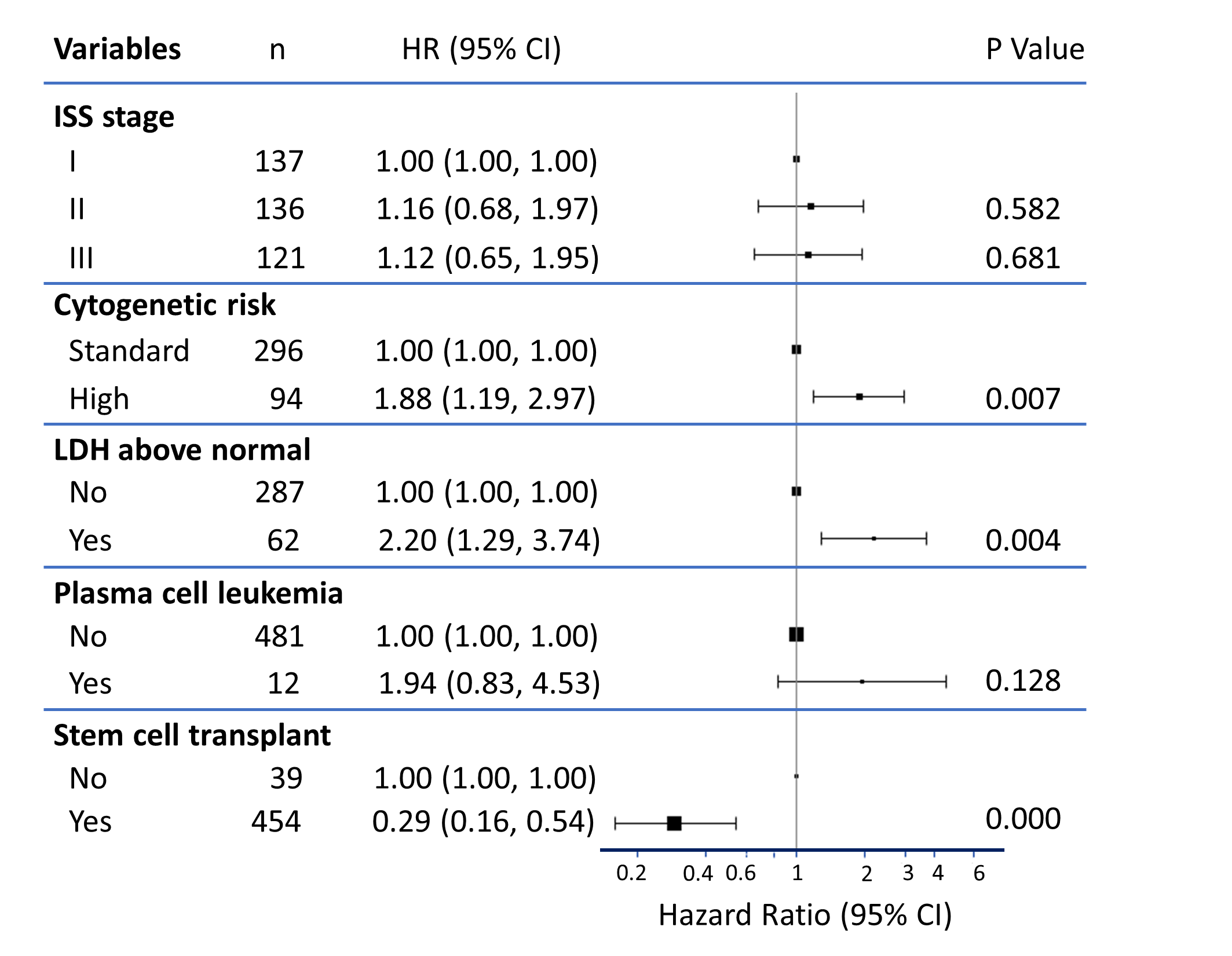
**

**Table S6b. Forest plot showing multivariable regression for the outcome of death after first line of treatment (with clinician specified covariates).**

**SUPPLEMENTAL FIGURES**

Number of patients : 493

Median age : 46

**Figure S1. Age distribution of the cohort.**

**
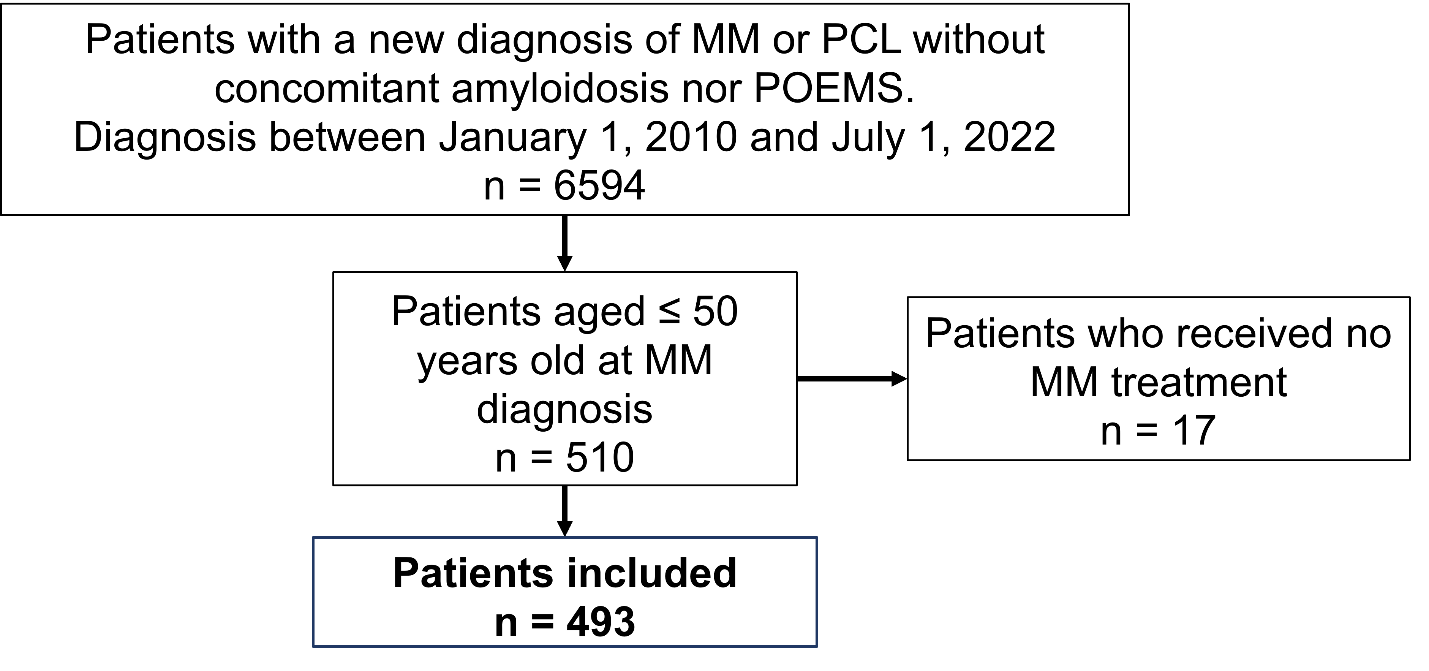
**

**Figure S2. Flow chart of patient inclusion from the CMRG database.**

CMRG: Canadian Myeloma Research Group; MM: multiple myeloma; PCL: plasma cell leukemia; POEMS: polyneuropathy, organomegaly, endocrinopathy, monoclonal component, skin changes.


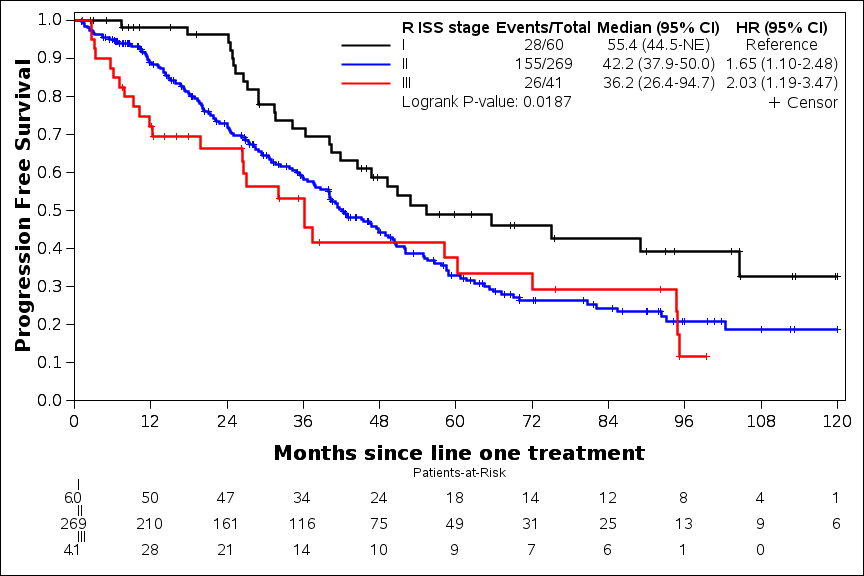


**Figure S3a. PFS based on R-ISS.**


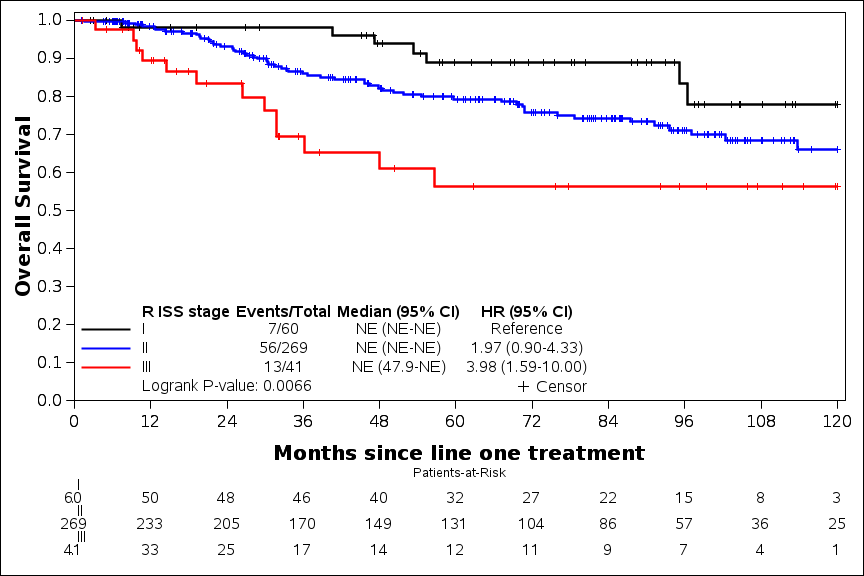


**Figure S3b. OS based on R-ISS.**


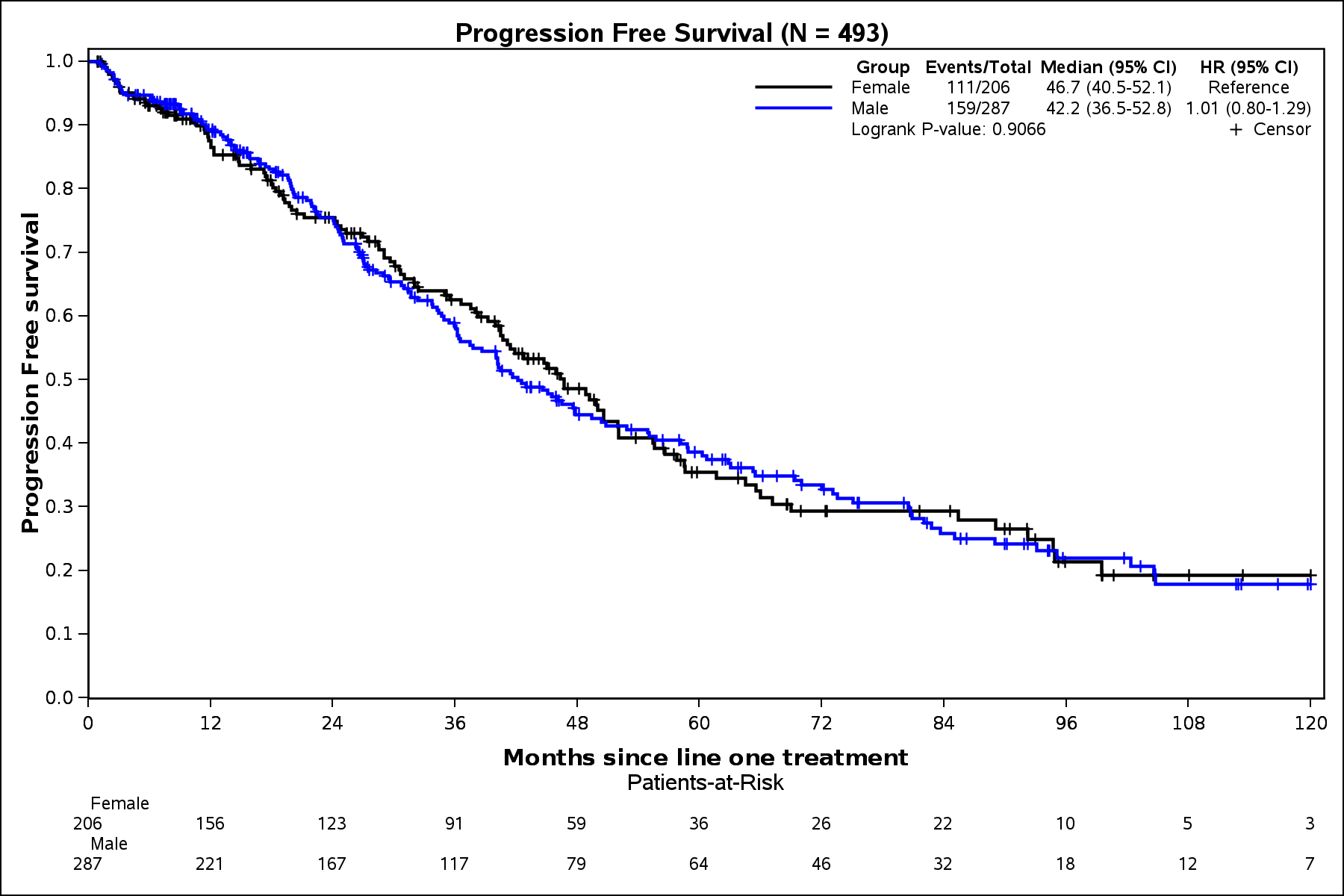


**Figure S4. PFS comparing female and male patients.**


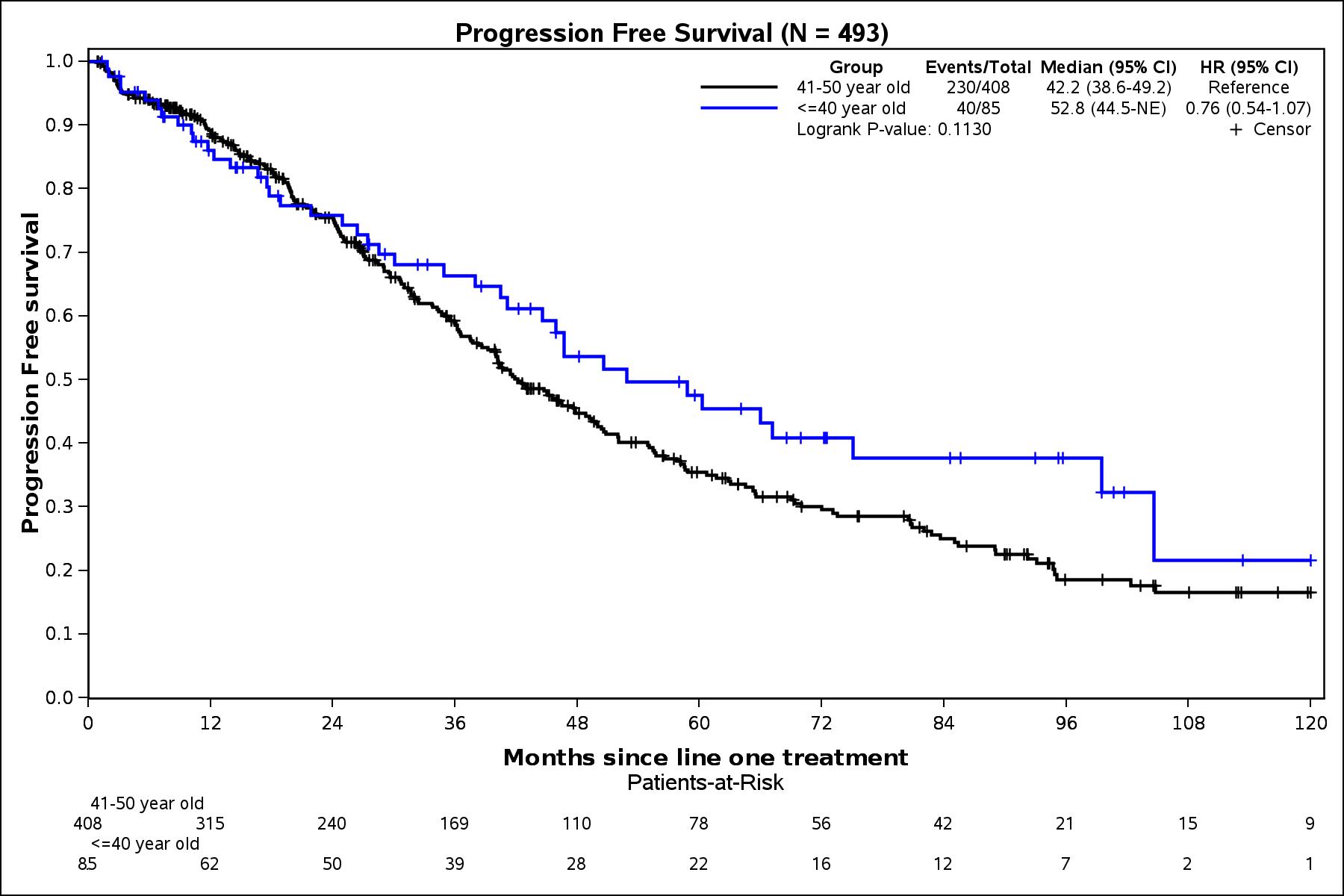


**Figure S5. PFS comparing age groups ≤ 40 versus 41-50 years.**
